# A validation of adult suicidal ideation questionnaire among Nigerian University students

**DOI:** 10.4314/ahs.v23i1.57

**Published:** 2023-03

**Authors:** Sunday Adeyemo, Olugbemi Olukolade, Afolabi Aroyewun

**Affiliations:** 1 Olabisi Onabanjo University, Psychology; 2 University College Hospital Ibadan, Department of Clinical Psychology; 3 University of Lagos, Department of Psychology

**Keywords:** Adult suicidal ideation questionnaire, psychometric properties, Nigerian University students

## Abstract

**Background:**

To prevent completed suicide among young adults in the university, assessment of suicidal ideation should be encouraged. This study aims to investigate the reliability, exploratory factor analysis, and validity of adult suicidal ideation questionnaire among Nigerian university students.

**Methods:**

Descriptive cross-sectional survey was the preferred choice of research design for this study utilizing convenience sampling technique to recruit participants. 6 universities divided into two equal numbers between federal-funded and state-funded ones in Nigeria were selected, and 2,702 students were sampled to fill the questionnaires. Instruments for data collection included Rosenberg Self-esteem questionnaire and Kessler Psychological distress scale to validate ASIQ. Ethical clearance for this study was collected from Olabisi Onabanjo University Teaching Hospital, Nigeria.

**Results:**

The results of the study showed that internal consistency of ASIQ was .951 while validity analyses proved that self-esteem and psychological distress diverged and converged respectively with the three subdomains of suicidal ideation as measured by ASIQ.

**Conclusion:**

This study concludes that ASIQ has slight modification from the original version among young adults in Nigeria. The scale is reliable and valid but as a 22-item instrument on a 5-point Likert scale.

## Introduction

Suicide is the end of a process that involves actions such as suicidal ideation, plans, and attempts of suicide and, likely completion of the action. It is a process that is burdensome to the survival and mental health of young adults. This period of development has been identified in studies centred on university students in Nigeria^1^, ^2^, ^3^. According to World Health Organization 4 suicide was reported to be the second leading cause of death among 15-29 agers. However, less is known about suicidal ideation especially in low and middle-income countries 5. Suicidal ideation is a risk factor for the whole issue of suicide 6. According to Reynolds 7, suicidal ideation is “thought and ideas about deaths, suicide, serious self-injurious behaviors, and thoughts related to the planning, conduct, and outcome (e.g., response of others) of suicidal behavior”. In other words, suicidal ideation is cognitively based. Suicidal ideation is a serious mental health condition in which, two out of three who experience it would make a suicidal plan and go ahead to attempt suicide 8. Before now, suicidal ideation was thought to be less common among young adults 9, but recent estimates have shown that about 15.2 % of university students in Ghana 10 and 21 % among Norwegian university students 11 reported the distressing thoughts of suicide.

Self-esteem and psychological distress are widespread concepts in the scholarship of psychological health with several definitions. More specifically, definitions of self-esteem emphasize the way people view themselves. Either as a positive or negative attitudinal evaluation of oneself 12 or as a way of giving approval or disapproval to oneself 13, self-esteem represents a behavioral notion about an individual. Psychological distress incorporates the features of depression and anxiety 14. Manifestation of psychological distress has a lot to do with the experience of stress which stretches one's ability to cope 15. Evidence has shown an association between self-esteem and suicidal ideation among adolescents and young adults^16^; ^17^; ^18^, and that psychological distress is related to suicidal ideation 19.

Much is desired to be known about suicide estimates especially in developing nations as information on suicide is scarce 20. In Nigeria, studies are yet to start focusing on suicidal ideation among university students. This could be a problem that is reminiscent of general suicidal rates in Nigeria such as strong taboo around suicide and stigma associated with its attempts 21, and; other reasons specific to university students such as what Hamaideh 22 described as “self-imposed stressors and pressures”.

Admittedly, nothing can be done to help when suicide finally happens, however, a lot can be done in order to plan for the prevention of suicidal ideation. Furthermore, the fact that university students are in a transition phase 23 in their lives shows how important it is that suicidal ideation should be identified early enough. Also, having a baseline of measurement to plot the graph of treatment gain is another important reason for the assessment of suicidal ideation. A search from extant literature by the authors revealed that much work has been done on suicidal ideation among secondary school students in Nigeria while Aloba, Adefemi & Aloba 24 focused on undergraduate students of Obafemi Awolowo University for the validation of positive and negative suicide ideation inventory.

The Adult suicidal ideation questionnaire (ASIQ) is an instrument designed to assess suicidal ideation specifically among university students. It measures the level at which individuals have been nursing the thoughts of killing themselves over a month period. It was developed by Reynolds 25 as a modification of the suicidal ideation questionnaire (SIQ) which was to measure suicidal ideation among adolescents. ASIQ is a 25-item self-report questionnaire that has been found to be reliable and valid. It has an internal consistency value of .97. The use of 25-item ASIQ as a self-report with Likert response format is more advantageous than that of close-ended response format as used in Amare, Woldeyhannes, Haile, & Yeneabat^20^. ASIQ has four domains, one of which contains one item. Psychometric properties for ASIQ have been shown among university students in some schools in Asia^26^, and among police officers in South Africa 27, however, the importance of this scale in the assessment of suicidal ideation needs to be established among young adults in a developing nation like Nigeria as its use can help in preventing completed suicide. Thus, this study was designed to provide evidence of reliability for ASIQ in Nigeria context, secondly to subject the scale to exploratory factor analyses which according to Watkins 28 is to ensure that orders and structures among measured variables are economically known and to determine the subdomains of the scale. Lastly, in evaluating the validity properties of the scale, we hypothesized that self-esteem would have a significant divergent validity value with ASIQ and psychological distress would have significant convergent validity value with ASIQ. The choice of self-esteem and psychological distress concept in the validity of ASIQ was so because of the high contiguity they have with suicidal ideation 25, 19.

## Methods

### Research Design

This study adopted a descriptive cross-sectional survey research design. This design was adopted to satisfy the factor that a particular sample can be drawn from a large population at a particular time and because of a high number of participants needed for the study.

### Sample Population

Six universities in southwest Nigeria were chosen using convenience sampling technique. This was informed because the southwest zone has more universities than the other zones, and there are more reports of suicide in the zone than elsewhere 29. Similarly, we conveniently recruited three federally funded and three state-funded universities, namely University of Ibadan in Oyo state, Obafemi Awolowo University, Ile-Ife in Osun State, and University of Lagos in Lagos State; Olabisi Onabanjo University, Ago-Iwoye in Ogun State; Adekunle Ajasin, Akungba-Akoko in Ondo State; and Ekiti State University. After that, students of the chosen universities were conveniently selected to respond to the questionnaires.

### Participants

A total of 2, 702 participants were recruited from the six above-listed universities. 1267(46.9%) males and 1435 (53.1%) females participated in the study. Meanwhile, participants whose age ranges between 16-20 years old were 1074(39.7%), 21-25years old were 1375(50.8%), 25-30 years old were 236 (8.7%), while above 30 years old were 20(7%) of the participants took part in the study. Religion of the participants revealed that about 2007(74.3%) were Christians, 618(22.9%) were Muslims while 77 (2.8%) were practitioners of African Traditional Religion. In our extensive search of the literature, we could not find any study that met this spread in relation to the number of participants and number of universities in Nigeria for suicide research.

### Procedure

The data were collected from August 2019 till January 2020 before covid-19 induced lockdowns in Nigeria. We obtained ethical approval from Olabisi Onabanjo University Teaching Hospital Research Ethical Committee before recruitment started. Sixty research assistants, ten in each of the universities were employed and trained on how to approach the participants and how to retrieve the questionnaires. Each of the authors supervised the collection of data in two of the universities which meant that the authors were in charge of 20 research assistants at the time of data collection. Ten research assistants attended one university and they were divided to cover the number of faculties in that particular university. The research assistants met the participants before classes and after classes and asked them to give their consent before they could accept to fill the questionnaires. Participants who did not give their consent were not recruited to participate in the study. The questionnaires were filled on the spot and retrieved immediately.

### Instruments

The questionnaire contains sociodemographic items such as gender, age, and religion affiliation of the participants. Rosenberg Self-Esteem Scale 9 was used to assess the participants' self-evaluation. Rosenberg self-esteem scale is a 10-item scale in which respondents express their degree of agreement on a 5-point Likert scale ranging from 1 ‘strongly disagree’ to 5 ‘strongly agree’. Five of the items (items 1, 2, 4, 6, 7) provide positive assessments of self-worth whereas (items 3, 5, 8, 9, 10) are measures of self-derogatory statements which indicate a reversal in their scoring pattern. A total raw score is computed by summing the scores across items. In this study, scores below 15 represent lower level of self-esteem, scores between 15 and 45 are normal whereas scores 46-50 represent a high level of self-esteem. The scale has a relatively high internal reliability (Cronbach alpha) of 0.76 among university students. This scale has been used fairly well in some Nigerian studies to measure similar concept as seen in Akanni & Oduaran and Tolulope, Olubukola & Olutayo 30, 31.

Another instrument used in the study was Kessler Psychological Distress Scale (KPDS) 32. It contains 10 items, each with a response set of 5 statements describing the global measure of distress based on questions about anxiety and depressive symptoms that a person has experienced in the past 4 weeks along a continuum from 1 (none of the time) to 5 (all of the time). A total raw score is computed by summing the scores across items. Results indicated high internal reliability (Cronbach alpha) of 0.90 among university students. Psychological distress has been shown to have a positive relationship with suicidal ideation when measured with KPDS 33.

Adult Suicidal Ideation Questionnaire (ASIQ) developed by Williams Reynolds was used to measure suicidal ideation among participants. The scale contains 25 items, each originally with a response set of 7 statements describing the severity of suicidal ideation in adults. The internal consistency was reported to be .97 and the validity measure was .86 (18); these values are accordingly acceptable 28. It is a scale that records scores on along a 7 continuum but we adopted a 5-response format from 1 (never) to 5 (always). A total raw score is computed by summing the scores across items (ranges from 25-125). Results indicated high internal reliability (Cronbach alpha) of 0.94 among university students.

## Results

### Results of the Validation of Adult Suicidal Ideation Questionnaire (Asiq)

The item total analysis precedes factor analysis of items in order to determine which items are consistent with others, while the inconsistent items are discarded. Thereafter, the consistent items are factored together to determine how many factors or construct they measure. The initial item-total analysis of all 25 items is presented in Table 1 below:

**Table I:** Showing Mean, Variance and Cronbach's Alpha using Item-total Statistics of the 25-item Suicidal Ideation Scale (SIS)

Items	X	SD	Scale Mean if Item Deleted	Scale Variance if Item Deleted	Corrected Item-Total Correlation	Cronbach's Alpha if Item Deleted
1. It will have been better if not alive	1.39	.85	40.86	285.310	.533	.941
2. I had thoughts of killing myself	1.54	.90	40.71	280.288	.674	.939
3. I have the thoughts of how to kill myself	1.51	.92	40.74	278.857	.709	.939
4. I have the thoughts of when to kill myself	1.46	.90	40.79	278.792	.727	.939
5. I am fond of writing suicide notes	1.38	.91	40.86	280.767	.651	.939
6. I like telling others of intent	1.71	1.06	40.54	281.739	.521	.941
7. I believe others will be happier if gone	1.58	1.00	40.67	277.565	.686	.939
**8. I know how others would feel**	**2.06**	**1.30**	**40.19**	**281.398**	**.421**	**.943**
9. I wish I were dead	1.51	.98	40.74	277.409	.708	.939
10. How easy it would be if dead	1.55	1.00	40.69	276.264	.727	.938
11. I think death will solve the problem	1.57	1.02	40.68	275.420	.736	.938
12. I think others are better off	1.80	1.12	40.45	275.688	.659	.939
13. I wish I had nerves	1.87	1.17	40.38	276.338	.609	.940
14. I wished I had never been born	1.62	1.07	40.63	275.405	.700	.939
15. Will die if I had chance	1.58	1.03	40.67	276.472	.695	.939
**16. Am not surprised the way people** **kill themselves**	**2.20**	**1.33**	**40.04**	**279.163**	**.462**	**.942**
17. I thought of killing myself but would not	1.76	1.18	40.49	275.738	.619	.940
18. I thought of having a bad accident	1.61	1.04	40.64	277.307	.668	.939
19. I believe life is not worth living	1.62	1.07	40.63	275.518	.696	.939
20. I believe life is too rotten to continue	1.66	1.07	40.59	274.617	.726	.938
21. I don't know the only way to be noticed	1.79	1.12	40.46	278.492	.581	.940
**22. I think others realize my worth**	**2.68**	**1.43**	**39.56**	**293.269**	**.124**	**.948**
23. No one cares if am alive	1.68	1.08	40.57	278.001	.615	.940
24. I will have, if I could kill myself	1.54	1.01	40.71	276.611	.709	.939
25. I will kill myself if things didn't improve	1.56	1.08	40.69	274.989	.705	.938

From the item statistical analysis in table, I above, the overall internal consistency of the scale is .942. Although, this Cronbach Alpha is above the acceptable standard of .50, however, the item-total correlation ranged from .124 to .736 for all the items, with average scale score variance of 301.417 and 42.25±17.36; if items 8, 16, 22 are deleted, the internal consistency of the scale would probably increase as these items (8, 16 and 22) are out-rightly inconsistent and invariant with the overall items of the scale. The improved version of the scale after items 8, 16, 22 was deleted and it is shown in the Table 2 below.

**Table II:** Showing an adjusted Item-Scale Correlation and Cronbach Alpha when items 8, 16 and 22 have been deleted from the SIS

Items	X	SD	Scale Mean if Item Deleted	Scale Variance if Item Deleted	Corrected Item-Total Correlation	Cronbach's Alpha if Item Deleted
1. It would have been better if not alive	1.39	.85	33.91	239.010	.535	.951
2. I had thoughts of killing myself	1.54	.90	33.76	234.389	.677	.949
3. I have the thoughts of how to kill myself	1.51	.92	33.79	232.923	.717	.949
4. I have the thoughts of when to kill myself	1.46	.90	33.84	232.710	.741	.948
5. I am fond of writing suicide notes	1.38	.91	33.92	234.318	.672	.949
6. I like telling others of intent to kill myself	1.71	1.06	33.59	236.241	.506	.951
7. I believe others will be happier if I'm gone	1.58	1.00	33.72	231.732	.694	.949
9. I wish I were dead	1.51	.98	33.79	231.392	.723	.948
10. How easy it would be if I'm dead	1.55	1.00	33.75	230.457	.739	.948
11. I think death will solve the problem	1.57	1.02	33.73	229.528	.752	.948
12. I think others are better off me	1.80	1.12	33.50	230.611	.648	.949
13. I wish I had nerves	1.87	1.17	33.43	231.483	.589	.950
14. I wished I had never been born	1.62	1.07	33.68	229.650	.711	.949
15. I will die if I had chance	1.58	1.03	33.72	230.572	.708	.949
17. I thought of killing myself but would not	1.76	1.18	33.54	230.612	.609	.950
18. I thought of having a bad accident	1.61	1.04	33.69	231.583	.673	.949
19. I believe life is not worth living	1.62	1.07	33.68	229.618	.712	.949
20. I believe life is too rotten to continue	1.66	1.07	33.64	228.973	.735	.948
21. I don't know the only way to be noticed	1.79	1.12	33.51	233.037	.574	.950
23. No one cares if I am alive	1.68	1.08	33.62	232.081	.623	.950
24. I will have, if I could kill myself	1.54	1.01	33.76	230.552	.727	.948
25. I will kill myself if things didn't improve	1.56	1.08	33.74	229.001	.725	**.948**

From the item statistical analysis in the above table II, the overall 22-items internal consistency measure increased to .951 from .942, this is excellently valid. Additionally, the correlated item total correlation increased and ranged from .506 to .752 across the items of the scale, with a scale mean of 35.30 and a standard deviation of 15.93 while the average scale variance of 253.85 and the total item of 22. In summary, from the above analysis, after deleting items 8, 16 and 22, the internal constituency of the scale ranges between .948 and .951

From the above Table III.1, a Principal Component Method which is a method used in extracting numbers of factors that account for a large proportion of variance of the total scale is used. The method uses the alpha coefficient loadings of each item when each is initially taken as a component, to factor together components that account for Eigenvalues greater than 1. Based on initial Eigenvalues greater than 1 and 25 for the maximum iteration for convergence, the result shows that about 3 factors were mainly extracted from the whole ASIQ. These 3 factors can be viewed as three different subscales/domains on a scale. It was observed that the first component which can be described as a state of hopelessness with statements like (I will kill myself if things didn't improve and I believe life is not worth living), accounts for a total initial eigenvalue of 11.08, thus explaining 50.39% of total ASIQ's variance. The second component which is more specific to the thoughts, plan of suicide is depicted with statements like (I have the thoughts of killing myself, I have the thoughts of how to kill myself), has a total of 1.14 eigenvalue, accounting for 5.21% of the total variation, while the third component talks more of what others would say if suicide happens. The third component accounts for 1.01 initial eigenvalue and accounts for 4.59% of total variation of the ASIQ. Therefore, these three components already accounted for a cumulative of 60.20% which is already more than half of the total ASIQ. From factor 1 through to factor 3, it was observed that they all accounted for a cumulative variance of 60.20% already. Other factors have relatively too weak eigenvalues to be reckoned which therefore were abandoned, which means items loading on them will eventually be discarded.

**Table III.1:** below shows the initial eigenvalues, number of extracted items, and sum of squared loadings if rotated for ASIQ

Component	Initial Eigenvalues			Extraction Sums of Squared Loadings		
	Total	% of Variance	Cumulative %	Total	% of Variance	Cumulative %
1	11.088	**50.399**	50.399	11.088	50.399	**50.399**
2	1.147	**5.212**	55.611	1.147	5.212	**55.611**
3	1.011	**4.596**	60.208	1.011	4.596	**60.208**
4	.850	3.863	64.071			
5	.760	3.456	67.526			
6	.652	2.966	70.492			
7	.612	2.782	73.274			
8	.584	2.654	75.929			
9	.535	2.434	78.362			
10	.494	2.247	80.609			
11	.481	2.186	82.796			
12	.467	2.122	84.917			
13	.443	2.014	86.931			
14	.422	1.917	88.848			
15	.384	1.745	90.593			
16	.378	1.717	92.310			
17	.345	1.566	93.876			
18	.302	1.374	95.249			
19	.294	1.336	96.585			
20	.274	1.244	97.829			
21	.242	1.101	98.930			
22	.235	1.070	100.000			

Extraction Method: Principal Component Analysis.

Although, the sums of squared loadings and their rotation in space show that out of these 3 factors, factor 3 is likely to be dropped because it has a loading less than the eigenvalue of 5, therefore probably making the ASIQ to be bi-dimensional. But the cumulative percentage for the scale will possibly be 45.49 which will be small. Therefore, in other to determine the consistency of each factor's loading in terms of how much it continues to account for in the scale total variation, the loadings are rotated, and from the rotated sums of squared loadings, the result shows that factor 1 – 3 still loads (60.20%) strong and account for above 60% of the total variation.

Additionally, the Kaiser Meyer-Olkin measure of sampling adequacy was .96. This shows that the sample of this study was greatly adequate and hence, items sufficiently measure each factor. The Barlett's test of sphericity was statistically significant also (p<.001; df =231; x2= 36131.26) which shows there were some relationships among the variables. Also, the determinant is 0.006 which shows that each item is not related to the extent of resulting in collinearity among the items as it is greater than 0.001 and greatly far away from zero.

From the Table III.II above, the result showed that three subscales were generated with the first containing 12 items including items 25, 24, 23, 19, 20, 15, 11, 18, 10, 9, 14, and 5; with the internal consistency ranges from .44 to .77. In the second subscale, 5 items were factored which include items 2, 3, 1, 4, and 17. The internal consistency of the second domain ranges between .51 to .81. The third domain contains 5 items including items 6, 13, 12, 21, and 7 with the internal consistency ranges between .48 to .80.

**Table III.II:** Showing Factor Loadings of Items on the Scale using the Principal Component Method of Extraction

Revalidated 22-Items for Suicidal Ideation Scale	Components			Communalities
	1	2	3	
25. I will kill myself if things didn't improve	**.777**	.248	.181	.698
24. I will have, if I could kill myself	**.764**	.284	.161	.691
23. No one cares if am alive	**.690**	.157	.200	.541
19. I believe life is not worth living	**.658**	.314	.247	.592
20. I believe life is too rotten to continue	**.648**	.265	.364	.622
15. Will die if I had chance	**.613**	.313	.310	.570
11. I think death will solve the problem	**.608**	.366	.346	.623
18. I thought of having a bad accident	**.605**	.308	.252	.525
10. How easy it would be if dead	**.588**	.397	.311	.601
9. I wish I were dead	**.570**	.471	.228	.598
14. I wished I had never been born	**.545**	.381	.334	.554
5. I am fond of writing suicide notes	**.440**	.385	.415	.513
2. I had thoughts of killing myself	.275	**.815**	.172	.769
3. I have the thoughts of how to kill myself	.316	**.778**	.238	.762
1. It will have been better if not alive	.171	**.705**	.150	.549
4. I have the thoughts of when to kill myself	.392	**.669**	.300	.691
17. I thought of killing myself but would not	.408	**.510**	.179	**.459**
6. I like telling others of intent	.094	.174	**.809**	.694
13. I wish I had nerves	.268	.239	**.650**	.550
12. I think others are better off	.354	.269	**.608**	.568
21. I don't know the only way to be noticed	.445	.059	**.563**	.518
7. I believe others will be happier if gone	.413	.386	**.488**	.558

Extraction Method: Principal Component Analysis. Rotation Method: Varimax with Kaiser Normalization.

Additionally, in Table III.II above, the commonalities table shows the initial commonalities before rotation, which shows the relationship between the variable and all other variables including the squared multiple correlation. From the result in Table 3.2 above, the commonalities among the variables are high (>.45).

From the above Table V, factor 1 (r [2702] =.951; p<.01), factor 2 (r [2702] =.854; p<.01) and factor 3 (r [2702] =.887; p<.01) significantly have high positive relationship with the overall suicidal ideation.

**Table V:** Showing the Convergent Validity between the Extracted Factors and a Different Scale (Psychological Distress) using Pearson Product Moment Correlations

Variables	Factor 1	Factor 2	Factor 3	Suicide Ideation	Psychological Distress		SD
Factor 1	1					18.89	9.42
Factor 2	.772**	1				7.67	3.80
Factor 3	.767**	.652**	1			8.75	4.07
Suicide Ideation	**.951** **	**.854** **	**.871** **	1		42.25	17.36
Psychological Distress	**.517** **	**.455** **	**.442** **	**.519** **	1	21.43	8.02

** Correlation is significant at the 0.01 level (2-tailed).

Moreover, factor 1 (r [2702] =.517; p<.01), factor 2(r [2702] =.455; p<.01), factor 3 (r [2702] =.442; p<.01) significantly have positive relationship with the psychological distress. Interestingly, the overall suicidal ideation (r [2702] =.519; p<.01) significantly has positive association with psychological distress.

Specifically, the convergent validity analysis established that psychological distress significantly has a convergent validity value with factor 1 at .517, factor 2 at .455, factor 3 at .442, and the composite score for overall suicidal ideation was .519. The results show that there are strong relationships between psychological distress and three domains of suicidal ideation as their respective convergent values were significantly above the standard of .40 Therefore, this result implies that the higher the suicidal ideation/thought of the participants, the similar increase is noted in the participant's psychological distress measure. Therefore, there is a significant convergent validity between suicidal ideation, its components, and psychological distress.

## Discussion

This study was designed to provide reliability properties for Adult Suicidal Ideation Questionnaire (ASIQ) among undergraduate university students in Nigeria; and also, to determine factor analysis of the scale through exploratory factor analysis while also conducting the validity values by investigating the relationship between self-esteem and suicidal ideation, and; psychological distress and suicidal ideation among university students in Nigeria.

We found out that ASIQ had an internal consistency value of .951 from 22-items. This is shorter by three items than the original version of Reynolds^25^. The deleted items failed to meet up with acceptable total correlation as proposed by Cohen^34^. We retained 22 items on a 5 Likert rating format. The reliability score of ASIQ among university undergraduates in Nigeria is slightly higher than the score among police officers in South Africa^27^. A similar validation study for a suicidal ideation instrument (Positive and Negative Suicide Ideation-PANSI-PI and PANSI-NI) measures both risk and protective factors of suicidal ideation as subscales of the instrument among 514 university students revealed internal consistency figures of .76 and .77 for the two subscales 24. Our study reported a higher internal consistency score than any of the three subscales identified. The point of difference between our study and that of 24 could be that while 24 was conducted among university undergraduate students in one university, we recruited our participants from six universities in the southwest geopolitical zone of Nigeria.

The evidence-based exploratory factor analysis disclosed ASIQ to be three-dimensional with adequate sample items and level of relationships among the factors. This is in tandem with the original version of the scale developed by Reynolds 25. The present study provides evidence of parsimonious measured variables and their subdomains following some of the steps enunciated by Watkins^28^ regarding the use of exploratory factor analysis.

Divergent validity was calculated between self-esteem and suicidal ideation. Self-esteem was found to inversely relate to all the three subdomains of suicidal ideation as measured by ASIQ. This is expected as the findings from the study of Wan et al. 18 opined that adolescents in China experienced less suicidal ideation especially if they have high self-esteem. Essentially, high self-esteem is a factor of strength for adolescents. In contrast, the study by Al-Shawashereh^17^ pointed out that the more self-esteem values a student has, the more thoughts of suicide.

This present study confirmed that there was a positive convergent validity score between psychological distress and all the subdomains of suicidal ideation as measured by ASIQ. University education impinges on many aspects of the existence of university students. The level of distress experienced by university students can lead them to suicidal ideation 6. Our result received support from the cross-cultural study of Eskin et al. 19. Psychologically distressed students are prone to worry 21. Going to university comes with a rollercoaster of emotions which when met with the demands of university lives can increase the level of worry which in turn increases the level of suicidal ideation of university students. The relationships of self-esteem and psychological distress to the measurement of ASIQ confirm that it is an instrument that is measuring what it is supposed to measure.

## Recommendation

Because young adults have been reported to be the second most at-risk individuals to commit suicide, it is pertinent to say that to forestall completed suicide, university mental health services need to engage more in routine suicidal ideation assessment. Despite the strength of large data that this present study can boast of, some cautions are in order when interpreting the data. The study was conducted in the south western states of Nigeria with 6 states out of 36 states in the federation. Attempting to generalize the results as representing Nigeria university undergraduate students should be done with caution.

## Conclusion

This study concludes that ASIQ which is a cognitively-oriented measure of suicidal ideation among university students has a slight modification from the original version among Nigerian undergraduate students. It is reliable and valid but as a 22-item instrument on a 5-point Likert scale.

## Figures and Tables

**Table IV:** Showing the Divergent Validity between the Extracted Factors and a Similar Scale using Pearson Product Moment Correlations

Variables	Factor 1	Factor 2	Factor 3	Suicide Ideation	Self Esteem		SD
Factor 1	1					18.89	9.42
Factor 2	.772**	1				7.67	3.80
Factor 3	.767**	.652**	1			8.75	4.07
Suicidal Ideation	**.951** **	**.854** **	**.871** **	1		42.25	17.36
Self Esteem	**-.104** **	**-.012**	**-.033**	**-.048** *	1	30.94	6.99

** Correlation is significant at the 0.01 level (2-tailed)

* Correlation is significant at the 0.05 level (2-tailed). N=2702
